# Bioinformatics Analysis of Persistent Dysregulated Expression of Genes Involved in HCV‐Induced Neurological Disorders and Liver Injuries After DAA Treatment Through Weighted Gene Co‐Expression Network Analysis

**DOI:** 10.1002/hsr2.71488

**Published:** 2025-11-16

**Authors:** Mohadeseh Zarei Ghobadi, Mohammad Amin Nooraniyan Esfehani, Shohreh Shahmahmoodi, Ahmad Nejati, Abolfazl Keshavarz, Katayoun Samimi‐Rad

**Affiliations:** ^1^ Department of Virology, School of Public Health Tehran University of Medical Sciences Tehran Iran; ^2^ Food Microbiology Research Center, School of Public Health Tehran University of Medical Sciences Tehran Iran

**Keywords:** chronic hepatitis C, direct acting antivirals, module‐trait analysis, neuropsychiatric disorders, sustained virologic responses, WGCNA

## Abstract

**Background and Aims:**

The molecular processes involved in the progression of neuropsychiatric and liver disorders in some patients who have achieved sustained virologic response after successful DAA treatment are still unclear. To understand these processes, we investigated alterations in the transcription patterns of genes associated with neural and immune functions after DAA therapy.

**Methods:**

A total of six microarray gene expression datasets related to patients who had received DAA treatment were downloaded from the Gene Expression Omnibus. Three groups comprising pretreatment and posttreatment CHC patients, as well as healthy subjects, were considered for the analysis. A weighted gene co‐expression network analysis was then performed to identify the gene groups (modules) implicated in chronic hepatitis C before and after DAA treatment. Differential gene expression (DEG) analysis and protein–protein interaction network (PPIN) analysis were then used to determine the major dysregulated genes before and after treatment.

**Results:**

The common genes identified between the DEGs and selected modules, as well as further PPIN analysis, revealed the non‐normalization of novel neural‐related genes, including IRF3, FYN, CFL1, TGFβ1, DPYSL2, CDK5, and GIT1, as well as novel immune‐related genes, including IκBα, CD14, IL‐1β, IRAK1, TBK1, and CEBPB, after DAA treatment.

**Conclusions:**

Our findings suggest that DAA treatment does not lead to the normalization of gene transcription patterns in CHC patients up to 6 months after HCV clearance. The non‐normalization of neuronal and immune gene expression, along with subsequent changes in the activity of related pathways, may contribute to the persistence or progression of HCV‐induced neuropsychiatric disorders and liver injuries after DAA treatment. The identified genes and their altered expression patterns provide novel insights into potential molecular mechanisms underlying disease progression following successful DAA therapy. Furthermore, these genes may serve as candidate biomarkers for monitoring disease progression or as potential targets for therapeutic intervention.

## 1 Introduction

The majority of HCV‐infected patients (70%) develop a chronic infection that enhances persistent inflammation with a constant induction of inflammatory mediators. It also accelerates the development of liver injuries such as fibrosis and a variety of extrahepatic manifestations such as neuropsychiatric disorders with alterations in expression and function of some immune and nonimmune mediators [1, 2].

Studies on the association of gene transcription pattern with neuropsychiatric disorders, which are reported in more than 50% of patients with chronic hepatitis C (CHC) are scarce [1, 3]. However, a limited number of studies have examined neuropsychiatric disorders in CHC patients before and after successful DAA therapy and mostly they have focused on patient‐reported outcomes and neuropsychiatric symptoms [4, 5]. Some of these studies show that after DAA therapy, neuropsychiatric disorders are potentially reversible whereas others report that they have partially improved [4, 5]. Despite the effectiveness of these reports, the mechanisms by which HCV is involved in neuropsychiatric disorders are still unclear. It appears that different and complex inteinteractions plays among HCV‐induced derangements are probably implicated in neuropsychiatric disorders. Some of these derangements may be due to the alterations in the expression of mediators produced by genes with neural functions. This can underlie changes in signaling pathways related to these mediators and/or interfere with neurotransmitter systems. These may contribute to the development of neuropsychiatric disorders similar to the effect observed with immune mediators such as pro‐inflammatory cytokines in CHC patients. In fact, the continuous activity of the immune system due to HCV infection activates some signaling pathways which lead to the release of pro‐inflammatory cytokines. These cytokines cause neurological changes that predispose to neuronal impairment and subsequent neuropsychiatric disorders [2, 3]. Therefore, additional studies are essential to examine the changes in the expression of mediators produced by genes with neural functions, as well as the signaling pathways in which these mediators are involved, during chronic HCV infection and after successful DAA treatment. Given the complexity of neuropsychiatric disorders linked to CHC, it is crucial to investigate how specific biomarkers can deepen our understanding of the underlying mechanisms of neurodegeneration, similar to findings reported in other neurodegenerative conditions [6, 7, 8].

Studies on the inflammatory cytokine environment in chronic HCV infection and after DAA therapy reveal significant alterations in cytokine expression in CHC patients [9, 10, 11]. While some research suggests that successful DAA treatment can restore cytokine levels and reverse immune exhaustion [9, 10], others indicate that these changes are not fully reversible, as only elevated cytokines decrease posttreatment without full normalization [9, 11]. Importantly, liver disease progression and HCC may still occur after achieving SVR, particularly in patients with advanced fibrosis or cirrhosis [12]. In addition, patients over 65 face a 0.95% annual HCC risk posttreatment, regardless of cirrhosis [13, 14]. These complications may partially be related to the non‐normalization of the expression pattern of immune mediators after successful treatment.

To better understand the pathogenesis mechanisms of HCV in neuropsychiatric disorders and liver injuries, particularly their progression after DAA therapy, we examined changes in mediator expression and activated signaling pathways. Our study aimed to uncover the molecular mechanisms by which these mediators function in disease pathogenesis [1, 10]. Using weighted gene co‐expression analysis, we identified co‐expressed gene groups involved in shared biological pathways. Our findings revealed new dysregulated genes implicated in neural and immune pathways in CHC patients and those who achieved SVR after DAA treatment.

## 2 Materials and Methods

### 2.1 Gene Expression Datasets Selection, Merging, and Preprocessing

**Figure 1:**
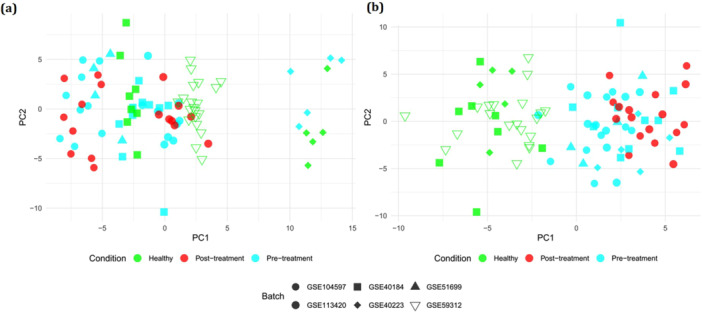
PCA plot (a) before and (b) after removing batch effect.

We explored Gene Expression Omnibus repository to detect the gene expression datasets related to HCV‐infected patients who had received DAA treatment. Based on our aim of analysis, we considered datasets reporting the expression data of samples before and after treatment (pretreatment and posttreatment). A total of six microarray datasets including GSE104597, GSE51699, GSE40184, GSE40223, GSE113420, and GSE59312 were identified for inclusion in our analysis. These datasets comprise 37 HCV‐infected samples before treatment, 17 HCV‐infected samples after treatment, and 33 healthy subjects. The details of the datasets are mentioned in Table 1. To remove the possible batch effect among different datasets, we used the remove BatchEffect function of the limma package (version 3.42.2) in the R environment (version 4.1.2) [15, 16]. The principal component analysis (PCA) plots before and after removing batch effect are depicted in Figure 1a,b. The expression data of healthy, pretreatment, and posttreatment samples were individually merged. The integrated data for each three mentioned groups were quantile‐normalized and log2‐transformed.

**Table 1:** Characteristics of the datasets included in the analysis.

Data set	Platform	No. of samples
GSE104597	Affymetrix Human Gene 2.0 ST Array	Pretreatment: 9
		Posttreatment: 9
GSE51699	Affymetrix Human Genome U219 Array	Pretreatment: 4
GSE40184	Affymetrix Human Genome U133A Array	Pretreatment: 10
		Healthy: 8
GSE40223	Illumina HumanHT‐12 V4.0 expression bead chip	Pretreatment: 5
		Healthy: 5
GSE113420	Affymetrix Human Genome U219 Array	Pretreatment: 9
		Posttreatment: 8
GSE59312	Affymetrix Human Genome U133 Plus 2.0 Array	Healthy: 20

*Note:* The top related pathways with a *p* value < 0.05 were considered for subsequent interpretations.

### 2.2 Weighted Gene Co‐Expression Network

The weighted gene co‐expression network analysis (WGCNA) was applied to identify the co‐expressed gene groups. For this purpose, the “WGCNA” package version 1.71 was applied in the R environment [17]. The constructed gene clusters (modules) may contribute to the same biological pathways. The first step to finding modules is determining the optimum value of soft‐thresholding powers β using “pickSoftThreshold” function. The *R* ^2^ ≥ 0.8 is commonly considered to opt β value. We selected *β* = 4. Afterward, an adjacency matrix is constructed using the calculation of Pearson correlation between each pair of genes. Next, the adjacency matrix is matched the scale‐free topology criteria using the optimum *β* value. Later, Topological Overlap Matrix (TOM) comprising the connectivity values of the gene network [18] and then dissimilarity TOM (dissTOM) are specified by transforming the adjacency matrix. In this study, we used unsigned network. An unsigned network in WGCNA is often used when the goal is to find groups of genes that behave similarly in terms of expression levels, regardless of whether they increase or decrease together.

Lastly, the “hclust” function and dynamic tree cut algorithm are employed to identify the modules by cutting the dendrogram branches constructed by hierarchical clustering. Modules with high similarity scores are then merged with a threshold value of 0.25 utilizing the mergeCloseModules function.

### 2.3 Construction of Module–Trait Association

To detect remarkably related modules to clinical traits including healthy, pretreatment, and posttreatment groups, the first principal component of a given module known as the module eigengene (ME), was calculated [17, 19]. The correlation between MEs and clinical traits was assessed by a two‐sided Pearson's correlation tests between MEs and clinical traits using “cor“ function and *p*‐value by “corPvalueStudent” function in the WGCNA package (version 1.71) [20]. The *p* value < 0.05 was considered to identify the meaningful correlations.

### 2.4 Identifying Differentially Expressed Genes (DEGs)

The DEGs between healthy and pretreatment as well as between healthy and posttreatment groups were determined using the Bioconductor package Limma (version 3.42.2) [21]. A Benjamini–Hochberg adjusted *p* value < 0.05 was used to control the false discovery rate and ensure statistical significance [22]. For healthy versus pretreatment comparisons, a |logFC| threshold of 1 (two‐fold change) was applied to capture meaningful gene expression changes related to active CHC infection. For healthy versus posttreatment comparisons, a more stringent |logFC| threshold of 2 (four‐fold change) was used to identify genes with persistent dysregulation after treatment. This choice was made by the study's aim to survey the molecular basis of non‐normalized transcription patterns following successful viral clearance. The higher threshold was selected to find genes with larger effect sizes, which are more likely to contribute to the persistence or progression of neuropsychiatric and liver disorders posttreatment.

### 2.5 Reconstructing Protein–Protein Interaction Networks (PPINs) and Enrichment Analysis

To reconstruct the intersection between genes at the protein level, the STRING database was employed [23]. To this end, the genes in the desired modules were submitted to STRING, and the possible interactions between protein levels were explored (medium confidence [0.4]). The PPINs [24] were visualized using Gephi (0.9.2) [25]. After visualizing the PPINs, enriched biological pathways associated with the members of these networks were identified utilizing ToppFun tools in the ToppGene webtool and EnrichR [26, 27]. The data being compared included the input gene list from the study, alongside predefined gene sets from established databases such as KEGG and Reactome [28]. Statistical significance was assessed using a two‐sided Fisher's exact test for EnrichR (https://maayanlab.cloud/Enrichr/) and a one‐sided hypergeometric test for ToppGene (https://toppgene.cchmc.org/help/publications.jsp), with *p* values < 0.05 considered statistically significant.

## 3 Results

### 3.1 Construction of Weighted Gene Co‐Expression Network (WGCN)

**Figure 2:**
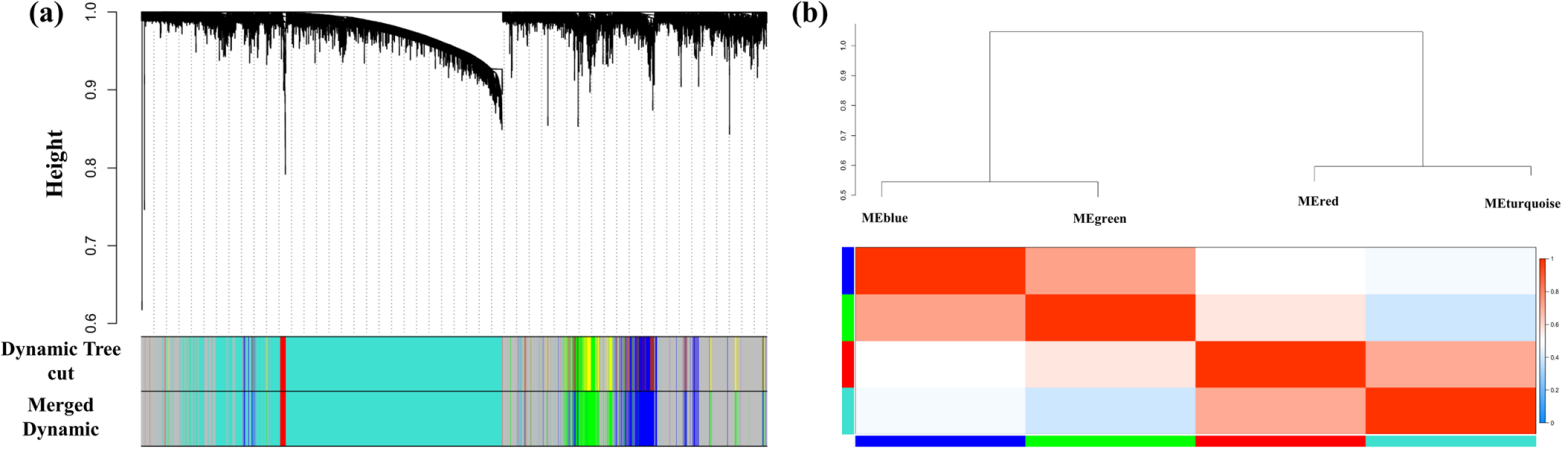
(a) Dendrogram of clustered genes based on a dissimilarity value (1‐TOM). The colored rows represent various modules obtained by the dynamic tree cut function and after merging similar modules. (b) The eigengene adjacency heatmap of the obtained modules. (c) The correlation between modules was characterized by the positive correlation (red) and negative correlation (blue) colors.

To construct a WGCN, the following steps were performed: calculating adjacency matrix and TOM dissimilarity, hierarchical clustering, cutting the branches of the dendrogram to identify the modules, and finally merging the similar ones. Figure 2a demonstrates the dendrogram with individualized modules, each denoted by a unique color. As illustrated, the dendrogram displays the hierarchical structure of gene relationships, with each module represented by a unique color for easy identification. In total, we identified four distinct modules; notably, the gray module consists of genes that do not belong to any specific module. Moreover, Figure 2b presents the final modules along with an eigengene adjacency heatmap that highlights the relationships among these modules [29]. To further confirm the distinctness of the blue and green modules, we calculated the Jaccard index using the R package GeneOverlap, finding no overlap between these modules (Jaccard index = 0, overlap count = 0). This complete separation is visually supported by the WGCNA dendrogram, where the blue and green modules form distinct branches with a significant clustering height difference, and by the ME correlation heatmap, which shows a near‐zero correlation (0.0) between MEblue and MEgreen, reflecting independent co‐expression patterns.

### 3.2 Identification of Modules Related to the Posttreatment Condition

**Figure 3:**
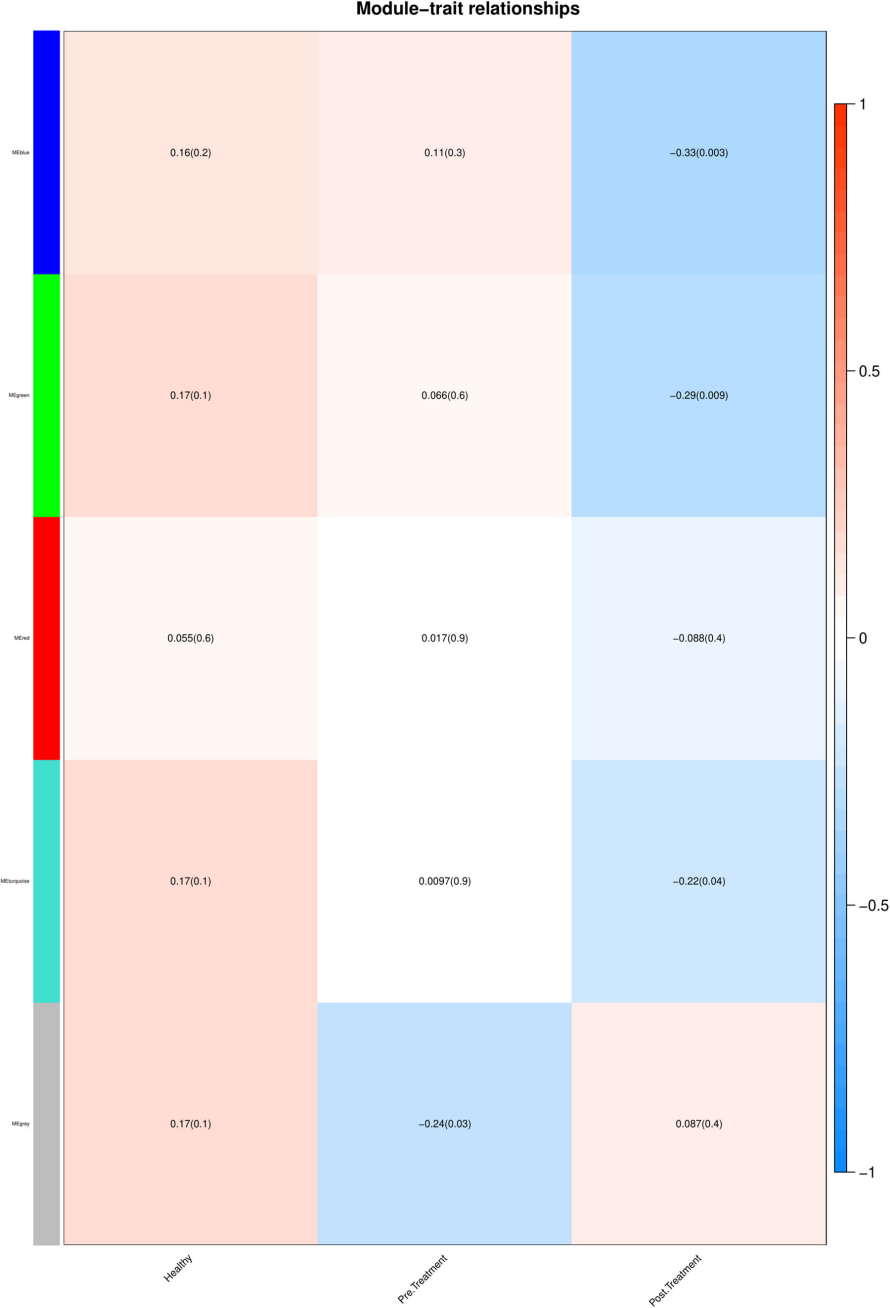
The module‐trait relationships. The correlation and *p* value between each condition (horizontal axis) and modules (vertical axis) are specified in distinct cells. Modules blue and green are remarkably correlated with the posttreatment conditions (*p* < 0.05 and and correlation> 0.25).

The module‐trait heatmap provides a visual representation of the correlations between the identified MEs and each studied group, highlighting the potential significance of the genes within each module in relation to specific traits. A higher correlation indicates a stronger association, suggesting that the genes in that module may play an important role in influencing the desired trait. Figure 3 indicates the module‐trait relationships in which the horizontal axis illustrates various conditions and the vertical axis represents the module names. The *p*‐value < 0.05 and correlation> 0.25 criteria determine the modules that are remarkably correlated with each condition [18]. From this view, modules blue and green are remarkably correlated with the posttreatment conditions (Supporting Information S1: File 1).

### 3.3 Determining DEGs and Reconstructing PPINs

**Figure 4:**
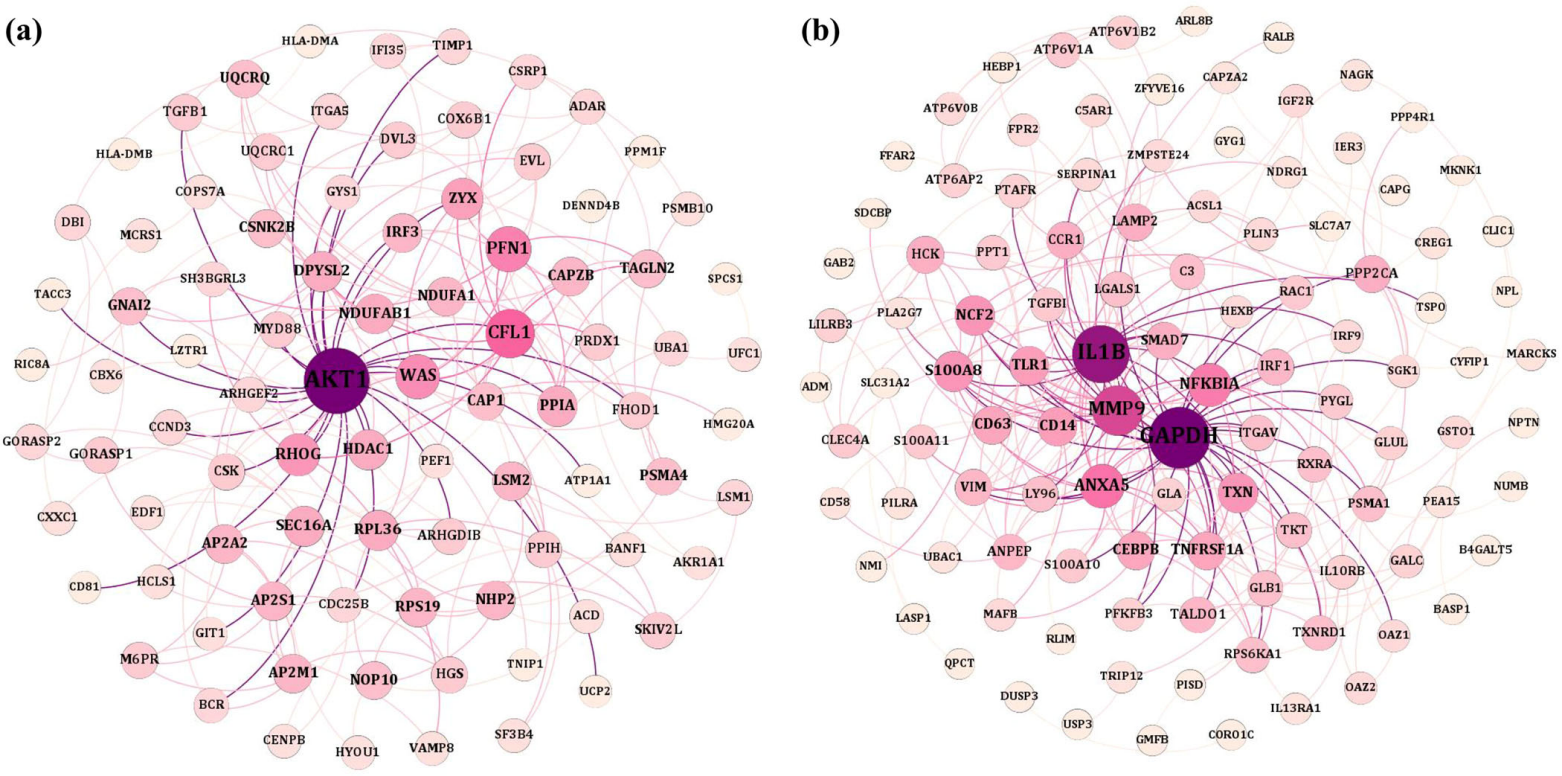
The PPINs for (a) blue and (b) green modules. The node size and color denote the degree of nodes since the higher size and intense color show a higher degree. AKT1, CFL1, NDUFAB1, PFN1, IRF3, ZYX, WAS, RHOG, and DPYSL2 ahave higher degree in blue module; and GAPDH, IL1B, MMP9, ANXA5, NFKBIA, SMAD7, S100A8, CCR1, and TLR1in green module.

The DEGs identified between the healthy and pretreatment groups (DEGs_H‐pre) as well as between the healthy and posttreatment groups (DEGs_H‐post) were systematically determined to understand the impact of HCV treatment on gene expression. A comprehensive list of these DEGs is provided in Supporting Information S2: File 2. Subsequently, we identified the common genes shared between DEGs_H‐post and DEGs_H‐pre, as well as those present in the blue and green modules, which are detailed in Supporting Information S3: File 3. To further explore the interactions among these common genes, we utilized the STRING database to reconstruct two PPINs, as illustrated in Figure 4. In these networks, nodes represent genes, and their sizes and colors correspond to their degree of connectivity. A larger and more intensely colored nodes indicate a higher degree of interaction with other proteins. Figure 4a highlights the PPIN of the blue module, where AKT1 emerges as the central hub with the highest degree of connectivity, followed by CFL1, NDUFAB1, PFN1, IRF3, ZYX, WAS, RHOG, and DPYSL2, all of which exhibit significant interactions within the network. Similarly, Figure 4b illustrates the green module, where GAPDH is the most highly connected protein, accompanied by IL1B, MMP9, ANXA5, NFKBIA, SMAD7, S100A8, CCR1, and TLR1, which also demonstrate high connectivity and play critical roles in the network.

### 3.4 Pathway Enrichment Analysis

**Figure 5:**
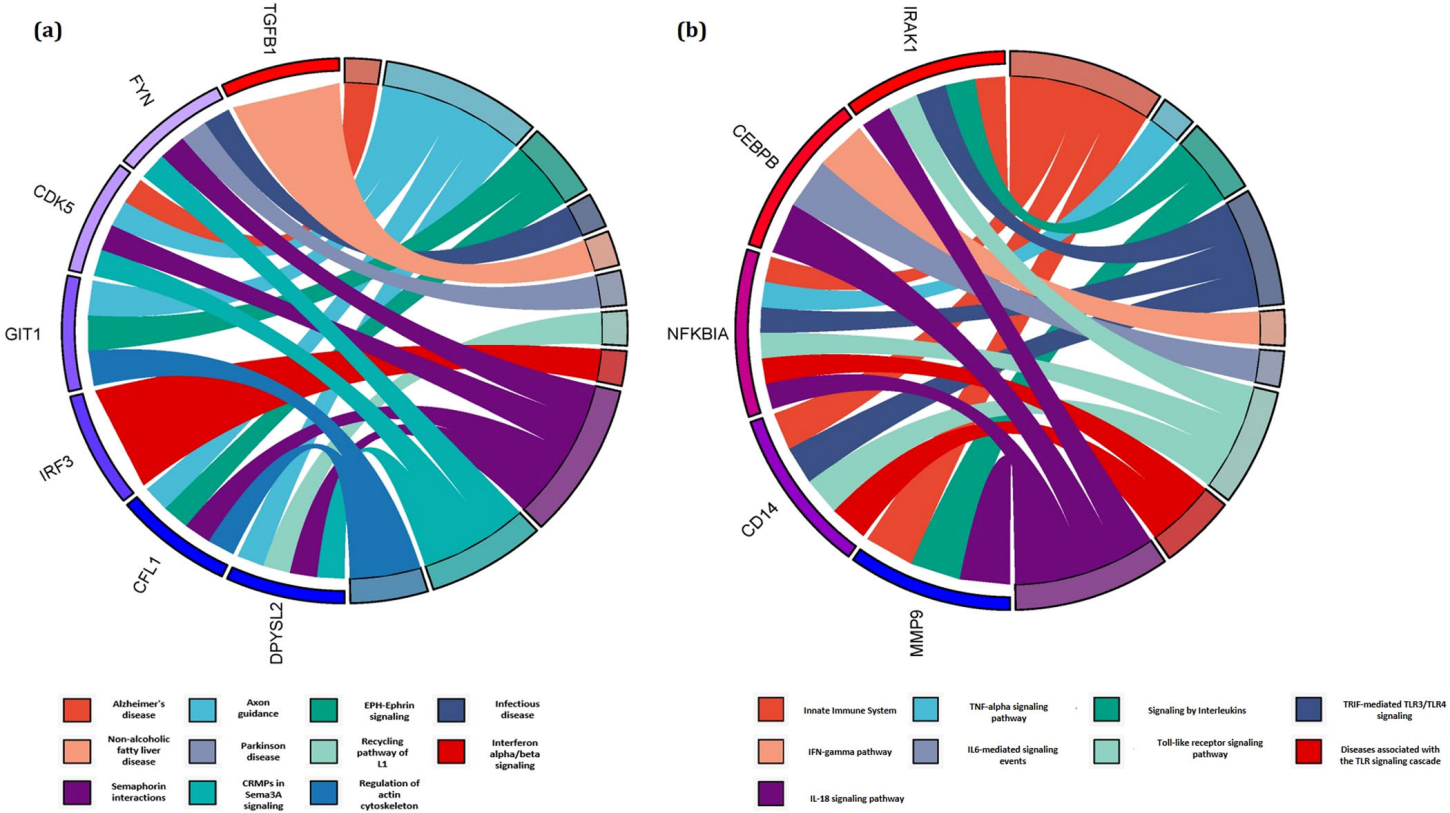
The enriched pathways by genes of (a) blue and (b) green modules.

The pathway enrichment analysis for the gene members of each blue and green modules were performed. The outcomes revealed that the genes belonging to the blue module are involved in Axon guidance, Infectious disease, Recycling pathway of L1, CRMPs in Sema3A signaling, Interferon alpha/beta signaling, Semaphorin interactions, EPH‐Ephrin signaling, Parkinson disease, Alzheimer's disease, Regulation of actin cytoskeleton, and nonalcoholic fatty liver disease (Figure 5a). Moreover, the terms including Innate Immune System, TRIF‐mediated TLR3/TLR4 signaling, IL6‐mediated signaling events, TNF‐alpha signaling pathway, IFN‐gamma pathway, Toll‐like receptor signaling pathway, Diseases associated with the TLR signaling cascade, Signaling by Interleukins, and IL‐18 signaling pathway were enriched by genes in green module (Figure 5b). The results specified this fact that the two PPINs were enrichrd in various neurological and immunological pathways after DAA treatment. These pathways were mostly enriched by IRF3, FYN, CFL1, TGFB1, DPYSL2, CDK5, and GIT1 in blue module; NFKBIA, MMP9, CD14, IRAK1, and CEBPB in green module. All of these genes are also among DEGs between healthy and pretreatment. Therefore, it may also confirm the effect of HCV infection in the occurrence of neurological disorders and liver injuries before and after DAA treatment.

### 3.5 The Possible Signaling Cascades in the Subjects After Posttreatment

**Figure 6:**
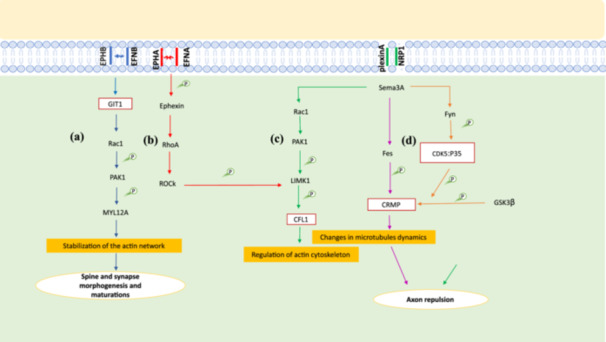
The proposed neurological signaling networks involved in neuropsychiatric disorder during chronic HCV infection and after DAA treatment. (a) After the binding of ENFB to EPHB and the phosphorylation of GIT1 in neurons, Rac‐1 is activated and phosphorylates the PAK‐1. The p‐PAK also phosphorylates MYL12A which promotes synapse anddendritic spine morphogenesis. (b, c) Activation of EPHA through binding of EFNA in neurons leads to phosphorylation of Ephexin1. The p‐Ephexin1 activates RhoA, Rock and LIMK1. The activated of LIMK1 stimulates the phosphorylation of CFL1, stabilization of actin cytoskeleton changes and axon repulsion similar to when Sema3A activates PAK1 and LIMK1 after binding to plexin A and NRP1. (d) Sema3A also activates Fes and Fyn. While activated Fes phosphorylates CRMP directly, the activated Fyn phoshphorylates CRMP by recruiting CDK‐5 in neurons. The p‐CRMP causes changes in microtubules dynamics which leads to axon repulsion.

**Figure 7:**
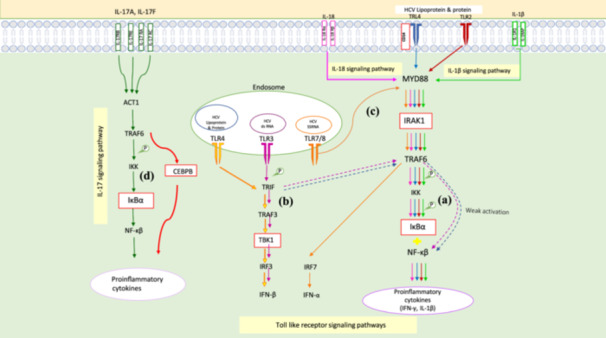
The proposed immunological signaling networks involved in liver diseases during chronic HCV infection and after DAA treatment. (a) In IL‐18, TLR2, TLR4 and IL‐1β after formation of complexes with their receptors and forming a complex with MyD88, IRAK1 associates with TRAF6. Then, with IκBα phosphorylation and its degradation, NF‐κB is released and translocated into the nucleus to generate signals which stimulate IFNγ and pro‐inflammatory cytokine production. (b) In TLR3 and TLR4 pathways located in the endosome, TRIF adapter protein mediates the induction of type 1 IFN genes through the recruitment of TBK1 and also activates NF‐κB slowly and generates pro‐inflammatory cytokines. (c) In TLR7 signaling, TLR7 acts as a sensor for HCV RNA, and MyD88 and TRAF6 like MyD88 and IRAK1 from a complex with IRF7 which causes to be translocated into the nucleus where it induces the expression of type 1 interferons. (d) In IL‐17 signaling pathways, through binding of IL‐17 to receptor complex and recruitment of ACT1 adapter, TRAF6 binds to it and activates NF‐κB and CEBPB that mediate the transcription of pro‐inflammatory cytokines genes.

Various pathways databases including KEGG and Reactome as well as enrichment results were employed to suggest possible signaling cascades in the patients after DAA treatment. The proposed signaling cascades involved in neuropsychiatric disorders during chronic HCV infection and following DAA treatment are illustrated in Figures 6 and 7 which widely discussed in the next section.

## 4 Discussion

In this study, we reported novel genes with neural and immune functions whose transcription patterns changed significantly in patients who achieved SVR after DAA treatment compared to CHC patients and the healthy group. The changed pattern was not normalized until 6 months after successful DAA therapy. This may be due to the effect of viral Ags on the nervous or immune system during chronic infection which continues even after treatment. Moreover, the observed changes may be due to the epigenetic events caused by the virus or the drug used for treatment. These reveal that alteration in the gene expression in CHC patients can be promoted and maintained for a long time after HCV clearance. Moreover, pathway enrichment analysis indicated that these genes were involved in nervous system, neurological disorders, formation of neural network, inflammation and immune response pathways.

The suggestion that chronic HCV infection can cause neuropsychiatric disorders comes from observing a range of nonspecific symptoms such as depression, fatigue, schizophrenia, bipolar effective disorder and decreased abilities in the areas of concentration and working memory that are frequently reported in patients with HCV infection [1, 5, 30]. In this study, the two modules identified before and after DAA treatment. These modules suggested the involvement of multiple novel genes for the first time including CFL1, GIT1, CDK5a, and CRMP2 and their signaling pathways which were related to the nervous system and neurological disorders including Eph‐ephrin signaling and semaphorin interactions.

In Eph‐ephrin signaling pathway (Figure 6a), GIT1 contributes to spine morphogenesis, synapse formation and regulation of synaptic transmission [31, 32]. Since these processes are critical for brain development, memory, learning and overall cognitive function, changes in them are strongly related to cognitive impairment [33]. On the other hand, alterations in these processes are also closely associated with depression through impairment of brain circuitry and neuroplasticity which are responsible for mood regulation and stress resilience [34, 35]. Moreover, disruption in spine and synapse morphogenesis and maturatiion, due to their effects on neural circuits can lead to anxiety [36]. Therefore, given the function of GIT1 in the morphogenesis and maturation of spine and synapses and the role of these processes in the nervous system, there is the possibility that the significant decrease of GIT1 leads to changes or disruption in these processes which are associated with neuropsychiatric symptoms as cognitive impairment, depression and anxiety. In this line, some studies suggested that GIT1 deficiency may lead to cognitive dysfunction in neuropscychiatric diseases [37]. In addition, they showed that GIT1 neural knockout mice had cognitive deficits in associative learning and working memory. Some other reports also showed GIT1 knockdown leads to neuropsychiatric disorders such as depression and attention deficit hyperactivity disorder (ADHD) [38, 39]. WON et al. [39] reported that GIT1 deficiency in mice causes ADHD‐like phenotypes through a reduction in Rac1 and PAK3 activities and limited inhibitory presynaptic input in neurons. Our findings also suggested that in addition to Eph‐ephrin signaling, semaphorin interactions are probably affected during chronic HCV infection and after DAA treatment. As shown in Figure 6b,c, in both Eph‐ephrin signaling and semaphorin interactions CFL1 is phosphorylated and loses its affinity for actin which leads to actin cytoskeletal changes and axon repulsion [40, 41]. In semaphorin interactions (Figure 6d), Sema3A also activates Fes and Fyn, which phosphorylate CDK5 and CRMP, leading to axon repulsion [42]. Since axon repulsion influences brain structure and function in adaulthood, dysregulation in its cues and pathways can lead to disorders as cognitive impairment (by contribution to synapse loss and inhibition of neuroplasticity [learning, memory, adaptation]), depression (by reducing synaptic connections in mood‐regulating circuits and neurogenesis), fatigue (by prevention of adaptive rewiring of neural networks and anxiety (by influencing the formation and plasticity of brain circuits [43, 44, 45, 46, 47]. These are symptoms observed in more than 50% of HCV infected patients who have neuropsychiatric disorders. In our study, we found a significant reduction in the expression of CFL1, CDK5 and CRMP2 genes. In line with our results, some studies have also reported reduced expression of these genes and association of this reduction with some neuropsychiatric disorders [48, 49, 50, 51, 52, 53]. Some studies, considering the role of CFL1 in the remodeling of the actin cytoskeleton in pre‐and/or post synaptic compartments and CDK5 in regulating synaptic plasticity (learning and memory), report a decrease in their levels and activities could have negative effects on the morphology and function of spines and synaptic plasticity and as a consequence on cognitive functions [48, 54]. On the other hand, another study also indicated that knockout of CRMP2 in mice leads to impaired emotional, social and cognitive behavior, mild impaired learning, altered anxiety level, and hyperactivity [55]. Moreover, multiple reports reveal an association between significantly decrease in CDK5 and CRMP2 and some neuropsychiatric disorders such as schizophrenia, ADHD and bipolar [49, 50, 51, 52, 53, 55]. Many patients with HCV infection have neuropsychiatric disorders with symptoms as cognitive impairment, depression, fatigue and anxiety which are very common symptoms in diseases like schizophrenia, ADHD and bipolar [49, 50, 51, 52, 53]. Therefore, it is suggested that the observed decrease in the expression of GIT1, CFL1, CDK5 and CRMP2 genes probably affects the pathways in which they are involved, as Eph‐ephrin signaling and Semaphorin interactions, and may ultimately lead to the reduced axon repulsion and altered spine and synapse morphogenesis and maturation (Figure 6). Subsequently, neuropsychiatric symptoms resulting from these changes may occur. Moreover, the significant reduction of these genes after treatment compared to before treatment and the control group is probably related to the non‐ improvement or partial improvement of neuropsychiatric symptoms after successful DAA treatment that some studies report [39, 48, 50, 51, 53, 55]. Since the suggestions about the association between changes in the expression of the genes GIT1, CFL1, CDK5 and CRMP2 and the development of neuropsychiatric disorders are based on information and evidence obtained from patients with neuropsychiatric disorders who did not have HCV infection, it is necessary that further experimental investigations be conducted to confirm this association in patients with hepatitis C before and after DAA treatment.

Due to the important role of inflammation as one of the mechanisms in the development of neuropsychiatric disorders, multiple studies have been conducted on several immune factors, particularly inflammatory cytokines. In this regard, Cacciarelli et al. [56] suggested an important role for the chronic activity of the immune system in cognitive disorders related to HCV. They reported that in HCV infected patients, upregulate of some cytokines such as IL‐2, IL‐4, IL‐10, and IFN‐y may persist for 20–30 years, which can cause continuous activation of microglia and changes in the blood barrier. On the other hand, it can indirectly affect brain function by transmitting signals from the vagus nerve or other neuronal pathways [56, 57, 58, 59]. In another study, Senzolo and colleagues suggested that chronic immune system activity leads to the continuous production of cytokines like IL‐1, IL‐4, IL‐6, and TNF‐α, which are responsible for neural changes underlying neurological impairment [60].

Most studies indicate that the strength of the immune response to HCV is the determining factor in either viral clearance or the establishment of chronic infection [61]. Since liver injuries in CHC are mainly caused by immunopathological processes, with identification of the molecular mechanisms of these events we can better understand how the liver damages progress to cirrhosis and HCC in some patients after DAA treatment. Toll‐like receptors are responsible for recognizing viruses and generating innate immunity by induction of type I IFN and proinflamatory cytokines [62]. Our results indicated a significant decrease in the CD14, IRAKI, IкВα and IL‐1β, mediators related to the TLR‐2, 4 and IL‐1β signaling pathways (Figure 7a), after treatment compared to before treatment. These reductions after DAA treatment probably affect these pathways and may downregulate the produced inflammatory cytokines such as IL‐1β. In this regard, several studies also report that the reduction in IRAKI level can decrease NF‐κB activity and expression of inflammatory cytokines including IL‐1β [63]. Given the role of IL‐1β, this may lead to a decline in the necessary and effective inflammatory responses, which allow persistent viral replication, disrupt liver tissue repair, and promote liver injury. In addition, IL‐1β downregulation may inhibit T‐cell activation and differentiation, which weaken cellular immune responses. Since cellular immunity is the main defense mechanism against HCV infection and cancer, its weakening may enhance progression of liver damages and tumor initiation which makes the patients susceptible to liver cancer [64, 65, 66]. On the other hand, in the TLR3 and TLR4 pathways located in the endosome (Figure 7b), TRIF adapter protein through the recruitment of TBK1 and in the TLR7 signaling pathway (Figure 7c), MyD88 and TRAF6 like MyD88 and IRAK1 through forming a complex with IRF7 induce the expression of type I interferons [62, 67]. In our study, the significant reduction of TBK1 and IRAK1 probably causes these pathways to fail to function properly and leads to a decrease in the production of IFN‐α/β. The decrease in IFN‐α/β in liver of patients with HCV infection may weaken antiviral signaling pathways and impairs the activation of immune cells and production of other antiviral molecules. These allow the virus to persist and cause chronic liver inflammation and liver injury progression [68]. IL‐18 as an interferon‐γ inducing factor, plays an important role in Th1 responses required for protection against HCV. In the IL‐18 signaling pathway (Figure 7a), IFNγ and proinflamatory cytokines are produced [69]. Our results indicated a significant decrease in the expression of IRAK1 and IкВα after treatment, the two important components of IL‐18 pathway that probably cause change in the expression of IFNγ. Therefore, considering the IFNγ function, this change may dysregulate effective T cell responses, impair liver injury repair and promote persistent viral replications and liver inflammation, which may ultimately lead to the progression of liver injuries and development of advanced forms of liver diseases [70, 71]. More over, our results also demonstrated a significant decrease in CEBPB and IкВα after treatment compared to before treatment and the control group. The effect of these reductions in IL‐17 signaling pathway (Figure 7d) may lead to a decrease in inflammatory responses and subsequently persistent viral replication and progression of liver damage [72]. It is worth mentioning that, to confirm the proposed suggestions regarding the impact of changes in the expression of genes CD14, IRAKI, IкВα, IL‐1β, CEBPB and TBK1 on the development and progression of liver damage, performing clinical assessments on patients before and after DAA treatment, as well as experimental studies to validate these genes, is necessary.

It should be noted that in line with our study, other researchers have also reported changes in some immune‐related genes in patients with chronic HCV infection. In this regard, Saravia et al. showed the level of some cytokines such as CCL‐3, CCL‐4, CXCL10, PAI‐1, FGFb, TGFβ, and IL‐10 which was increased during HCV infection (80.7% had cirrhosis), significantly decreased after DAA treatment, and reached levels similar to healthy controls, while other factors such as suPAR and CCL11 did not change. They also found that serum levels of growth factors such as FGFb, PDGF‐BB and VEGF decreased more as the condition progressed from liver fibrosis to compensated cirrhosis and then to decompensated cirrhosis [10]. In another study, Hengst et al. [9] reported that although a significant decrease in the upregulated cytokines TRAIL, IL‐18, IL‐12p40, IFN‐a2, LTA, and IP10 was observed after DDA treatment, the titers of these cytokines did not normalize. They also showed that all six cytokines that had significantly decreased before treatment, remained at low levels up to 36 weeks after treatment. Recently, Jilkova et al., indicated that the levels of IL‐4 and IL‐13 were significantly higher, and the levels of 4‐1BB and PDL2 were significantly lower, in patients who developed HCC after achieving SVR with DAA treatment compared to those who did not. The researchers noted that IL‐13 and IL‐4 are associated with carcinogenesis and play a critical role in immune mechanisms leading to HCC. Moreover, epigenetic changes caused by HCV and the patients' conditions such as the severity of symptoms during chronic infection, the drugs used, age, and sex can reprogram host gene transcription and expression patterns. There are reports that show this reprogramming in liver diseases activates some pathways which are involved in the HCC development. Since epigenetic changes persist after HCV clearance with DAA treatment alteration in gene expression and signaling pathway activation also continue. These are in agreement with our results that normalization of expression of our genes was not observed after DAA treatment. Therefore, persistence of changes in the neuronal gene expression after DAA treatment in our study, can be one of the reasons for the non‐improvement of some symptoms reported in patients with neuropsychiatric disorders [60, 73, 74]. In addition, multiple studies have demonstrated the influence of demographic characteristics (age, gender, ethnicity, BMI, and socioeconomic status), comorbidities, and different DAA treatment regimens on cellular gene expression in chronic HCV infection [75, 76]. It is noteworthy that the effects of DAA treatment regimens not only lead to viral clearance but also result in significant changes in cellular gene expression, particularly genes associate with immunity and cellular responses [77]. Although we utilized publicly available datasets in our study which limited our effect on reducing the confounding factors mentioned above, most used datasets had considered demographic characteristics and comorbidities in their inclusion and exclusion criteria to minimize the impact of confounding factors as much as possible [78, 79, 80].

Overall, this study identified novel genes with neuronal and immune functions which remained dysregulated up to 6 months after treatment of patients with DAAs. We described that these genes involved in pathways related to nervous system, formation of neuronal network, neurological disorders, inflammation and immune system. However, further research must be conducted for; i) experimental validation of these genes; ii) confirmation of their precise functions in the pathogenesis of HCV infection and after DAA treatment; iii) using them as biomarkers to detect the risk of progression of neuropsychiatric and liver disease in patients after DAA treatment.

## 5 Conclusion

In the present study, for the first time of our knowledge, some novel neural and immune‐related genes with significant changes in their expression were identified in HCV chronic infection and after successful DAA treatment. They could be candidate biomarkers for disease progression monitoring and as effective targets for prevention or therapeutic approaches for patients who achieved SVR after DAA treatment but persistence or progression of neuropsychiatric and liver manifestations is still observed. Our findings also suggested that some neural and immune pathways were affected due to the dysregulated genes which can explain a part of the mechanism of HCV pathogenesis underlying neuropsychiatric and liver disorders after DAA treatment. Moreover, our results showed that a period longer than 6 month is required to normalize the expression of dysregulated genes. However, further experimental studies and models are needed to validate these preliminary results and confirm the exact role of dysregulated genes and related pathways in patients after HCV clearance with DAA treatment.

## Author Contributions

**Mohadeseh Zarei Ghobadi:** formal analysis, methodology, software, writing – original draft, writing – review and editing. **Mohammad Amin Nooraniyan Esfehani:** investigation, methodology. **Shohreh Shahmahmoodi:** data curation, validation. **Ahmad Nejati:** data curation, validation. **Abolfazl Keshavarz:** formal analysis, investigation. **Katayoun Samimi‐Rad:** conceptualization, writing – original draft, writing – review and editing, project administration, supervision.

## Conflicts of Interest

The authors declare no conflicts of interest.

## Transparency Statement

The lead author Katayoun Samimi‐Rad affirms that this manuscript is an honest, accurate, and transparent account of the study being reported; that no important aspects of the study have been omitted; and that any discrepancies from the study as planned (and, if relevant, registered) have been explained.

## Supporting information


**Supplementary Data File 1.** List of genes in modules blue and green.

**Supplementary Data File 2.** The differentially expressed genes between healthy and pre‐treatment (DEGs_H‐pre) samples as well as between healthy and post‐treatment groups (DEGs_H‐post).

**Supplementary Data File 3.** Common genes between DEGs_H‐post as well as DEGs_H‐pre and genes in modules blue and green.

## Data Availability

The authors confirm that the data supporting the findings of this study are available within the article [and/or] its supplementary materials.
